# Evaluation of Surface Roughness, Color Stability, and FTIR Characterization of Different Inorganic-Filled PEEK Materials After Coffee Thermal Cycling

**DOI:** 10.3390/polym18182264

**Published:** 2026-09-17

**Authors:** Aybike Cengiz Dağtekin, Samet Tekin

**Affiliations:** Department of Prosthodontics, Faculty of Dentistry, Fırat University, 23119 Elazig, Türkiye; s.tekin@firat.edu.tr

**Keywords:** CAD/CAM polymers, polyaryletherketone (PAEK), hydrothermal degradation, thermocycling, in vitro aging, spectrophotometric analysis

## Abstract

Inorganic fillers are incorporated into polyetheretherketone (PEEK) to enhance mechanical performance, yet their long-term stability in chromogenic media remains insufficiently explored. This study evaluated the color stability, surface roughness, and chemical changes in modified PEEK variants following coffee thermal cycling. In this study, four PEEK materials were tested: unfilled (P), glass fiber-reinforced (GFP), nanoceramic-filled (CR), and titanium dioxide-filled (TP). A total of 40 disk-shaped specimens (10 × 2 mm, *n* = 10) were prepared and subjected to 30,000 cycles (5–55 °C) in coffee. Color differences (ΔE) after 5000 and 30,000 cycles, roughness changes (ΔRa), and chemical alterations via qualitative Fourier-transform infrared (FTIR) spectroscopy were evaluated. Thermal cycling significantly affected all materials (*p* < 0.05). TP demonstrated superior stability with the lowest roughness increase (ΔRa = 0.017 ± 0.003 μm) and minimal discoloration (ΔE = 1.66 ± 0.25), remaining within the clinically acceptable threshold (ΔE < 3.3). Conversely, GFP showed the greatest degradation (ΔRa = 0.133 ± 0.008 μm; ΔE = 8.45 ± 0.34). FTIR confirmed no chemical backbone cleavage across groups. Filler type significantly dictates thermochemical resistance in PEEK. While titanium dioxide reinforcement preserves surface smoothness and color stability under in vitro aging, glass fiber reinforcement markedly accelerates staining and roughening. This study highlights the necessity of considering hydrothermal aging resistance and microstructural filler stability when selecting PEEK biomaterials for long-term clinical restorations.

## 1. Introduction

In modern prosthetic dentistry, the selection of an optimal framework or restoration material relies on the simultaneous achievement of biocompatibility, physical strength, and long-term optical success. Historically, polymethyl methacrylate (PMMA)-based resins, dental composite systems, and monolithic zirconia have been widely preferred as gold standards in various fixed and removable prosthetic applications [1,2]. However, conventional PMMA-based polymers exhibit drawbacks such as water sorption, structural fatigue occurring under intraoral conditions, and loss of color stability over time. These limitations pave the way for clinical fractures in the material under masticatory loads [3]. Although monolithic zirconia offers superior wear resistance and excellent color stability, its high elastic modulus can lead to stress shielding. This phenomenon may result in localized bone loss around supporting teeth or implants, as well as causing significant abrasive wear on antagonist natural teeth [4].

Computer-aided design and computer-aided manufacturing (CAD/CAM) blocks polymerized under industrial conditions provide higher structural homogeneity compared to resin materials prepared using conventional methods. These microstructural advantages render the mechanical performance of the material more predictable and directly enhance its clinical reliability [5,6]. In clinical settings, restorative materials are exposed to a complex intraoral environment that encompasses cyclic occlusal forces, staining solutions, continuous moisture, and thermal variations. In the long term, these environmental and mechanical factors have the potential to adversely affect the microstructural integrity and surface characteristics of the materials [7,8]. Among CAD/CAM polymers, polyetheretherketone (PEEK) stands out in prosthetic dentistry due to its superior properties. Belonging to the polyaryletherketone (PAEK) family, this semi-crystalline thermoplastic polymer exhibits high chemical stability, corrosion resistance, and excellent biocompatibility. Unfilled PEEK exhibits a lower elastic modulus (3–4 GPa) compared to conventional metallic alloys (110–130 GPa), thereby reducing stress shielding; moreover, when reinforced with inorganic fillers or fibers, its modulus can be tailored closer to that of cortical bone and dentin (12–18 GPa) [9].

Prefabricated PEEK blocks processed via 5-axis milling configurations suitable for CAD/CAM technology offer minimal residual monomer levels and reduced internal porosity. This dense molecular architecture allows the material to remain chemically inert and enhances its hydrophobic character. Consequently, modified or unfilled PEEK variants exhibit significantly lower staining susceptibility and lower initial surface roughness values compared to conventional PMMA and hybrid resin systems [10].

The color stability of prosthetic materials is directly related to the surface roughness and surface free energy resulting from the applied surface finishing procedures. Numerous studies in the literature have demonstrated a positive correlation between increased surface roughness and the discoloration susceptibility of restorative materials [11,12]. Thermal aging is one of the most frequently applied in vitro methods to simulate complex intraoral environmental fluctuations. Repeated temperature changes can lead to hydrothermal degradation in polymer-based materials through mechanisms such as water diffusion, matrix plasticization, and interfacial breakdown in multi-phase systems. These processes have the potential to adversely affect the optical properties, surface integrity, and mechanical stability of restorative materials over time. Therefore, investigating current CAD/CAM modified PEEK polymers is of critical importance for predicting the long-term clinical performance of these materials [8]. Coffee is a major source of caffeine that is widely consumed worldwide and constitutes an integral part of daily dietary habits [13]. Despite its hydrophobic structure, coffee has been reported to exhibit a high degree of adsorption onto the surface. According to the literature, 5000 thermal cycles of coffee aging between 5 °C and 55 °C are equivalent to six months of coffee consumption [14].

In summary, understanding the long-term physical and aesthetic performance of structurally modified PEEK-based polymers under various intraoral dietary conditions plays a pivotal role in determining clinical longevity compared to conventional restorative materials. Although studies exist regarding the color changes and optical behavior of unfilled and ceramic-filled PEEK systems, a distinct research gap remains in the literature concerning the long-term effects on the surface characteristics, roughness stability, and color stability of contemporary compositions, such as titanium dioxide-filled and glass fiber-reinforced PEEK, following chronic thermochemical exposure induced by globally consumed staining agents like coffee. Investigating the regulatory effect of different inorganic additives on material surface behavior is essential for the sustainability of restorative success. Therefore, the novelty of our study stems from conducting a detailed and comparative analysis of both unfilled and inorganic additive-containing PEEK variants following a simulated long-term aging protocol. In our previous investigation [15], the stainability and surface roughness alterations of modified PEEK variants were documented under static immersion across multiple dietary beverages, identifying coffee as the primary chromogenic medium inducing significant discoloration across neat and nanoparticle-modified PEEK matrices alongside severe surface alterations. However, static immersion models cannot replicate the severe intraoral hydrothermal stress generated by repetitive thermal expansion and contraction cycles. Building upon our earlier static findings [15], the present study was designed to bridge this critical knowledge gap by subjecting these inorganic-filled and unfilled PEEK variants to an extended dynamic hydrothermal aging protocol (30,000 coffee thermal cycles, equivalent to approximately 3 years of clinical service).

The aim of this study was to evaluate the color stability, surface roughness, and chemical changes in different modified PEEK materials subjected to standard surface treatments, both in the short and long-term following thermal cycling with coffee. The null hypothesis was that thermal aging would not lead to statistically significant differences in terms of color stability, surface roughness, and chemical structure among the tested modified PEEK materials.

## 2. Materials and Methods

### 2.1. Specimen Preparation

The sample size was calculated using G*Power software (version 3.1.9.7; Heinrich-Heine-Universität Düsseldorf, Germany) for one-way analysis of variance (ANOVA) across four independent groups. Based on an alpha level of α = 0.05, a statistical power (1-β) of 0.80, and a large effect size (f = 0.55), the minimum required sample size was determined to be *n* = 10 specimens per group (total *N* = 40).

A total of 40 disk-shaped specimens (10 × 2 mm, *n* = 10) were prepared from four different PEEK materials. The materials were sectioned to appropriate dimensions using a precision cutting machine (Struers Minitom; Struers, Copenhagen, Denmark). To standardize the specimen surfaces, they were ground under water cooling using 400, 800, and 1200-grit silicon carbide abrasives, respectively. Finally, all specimen surfaces were polished using a low-speed handpiece (KaVo Dental, Biberach, Germany) operating at a constant rotational speed of 10,000 RPM with a soft goat-hair brush and universal polishing paste (Ivoclar Vivadent, Schaan, Liechtenstein). Polishing was performed under standardized moderate manual pressure (approximately 2 N) for 30 s per specimen surface by a single calibrated operator to ensure maximum reproducibility. They were ultrasonically cleaned for 10 min, cleaned with 98% ethyl alcohol, and dried. The commercial names, compositions and manufacturer details of the evaluated PEEK materials are summarized in Table 1. The experimental groups and abbreviations used throughout the study are presented in Table 2. Filler integration was verified chemically through characteristic infrared absorption bands (Ti-O and metal-oxide lattice modes at 700–400 cm^−1^ for TP and CR; Si-O-Si at 1050–1000 cm^−1^ for GFP) on FTIR spectroscopy.

Because commercially standardized dental-grade CAD/CAM milling discs with 30% glass fiber content are currently not widely available in routine dental marketing, standardized glass fiber-reinforced formulations (such as TECAPEEK GF30) are commonly employed in dental and biomaterials research as representative models to evaluate the behavior of fiber-reinforced PAEK matrices in prosthodontics and implantology [15,16,17]. In our study, TECAPEEK GF30 was specifically included to assess and compare the surface degradation, roughness alteration, and color stability of an inorganic fiber-filled configuration alongside unfilled, nanoceramic-filled, and TiO_2_-filled formulations under chronic thermochemical exposure.

### 2.2. Baseline Surface Roughness and Color Measurements

The baseline surface roughness (Ra0) of each specimen was recorded using a contact profilometer (Mitutoyo SJ-410, Mitutoyo Corporation, Kawasaki, Japan) featuring a 2 μm diamond stylus tip radius, a measuring force of 0.75 mN, and a vertical resolution of 0.01 μm. In accordance with International Organization for Standardization (ISO) 4287 standards [18], measurements were performed using a Gaussian filter with a cut-off wavelength (λc) of 0.8 mm, an evaluation length of 4.00 mm (total traverse length: 5.5 mm), and a constant traversing speed of 0.5 mm/s. Three parallel traces were recorded across the center of each specimen, and their arithmetic mean was calculated as the representative Ra value.

A digital spectrophotometer (VITA Easyshade V, Vita Zahnfabrik, Bad Säckingen, Germany) was used to measure the L0*, a0* and b0* color parameters of each specimen according to the CIE (Commission Internationale de l’Éclairage) system. To standardize the evaluation position and avoid ambient light interference, the probe tip was positioned at a 90-degree angle to the specimen surface. All measurements were conducted under D65 illumination conditions at a constant distance against a non-reflective gray background. For each specimen, measurements were repeated across 3 different regions, and the average values were calculated and recorded as L0*, a0* and b0*.

### 2.3. Coffee Thermal Cycling Protocol

Following the initial color and roughness measurements, all specimens were thermally aged using a coffee solution (Nescafé Classic, Nestlé, Vevey, Switzerland) at temperatures ranging between 5 °C and 55 °C, in accordance with ISO 11405 guidelines [19]. The aging process utilized a dwell time of 30 s and a transfer time of 10 s. With a dwell time of 30 s in each bath (60 s per cycle), the cumulative immersion time in the coffee solution was 83.3 h (~3.5 days) for 5000 cycles (T1) and 500 h (~20.8 days) for 30,000 cycles (T2). The coffee solution was prepared according to the manufacturer’s instructions, using a ratio of 1 tablespoon of coffee to 177 mL of boiling water, and both the cold (5 °C) and hot (55 °C) containers were filled with the standardized coffee solution and refreshed every 12 h to maintain consistent chromophore concentration and pH throughout the aging process [20,21,22]. Coffee was specifically selected as the aging and staining medium in the present dynamic protocol based on our previous comparative investigation [15], wherein coffee demonstrated the most pronounced chromogenic potential and surface interaction among various tested dietary beverages under static conditions. In contrast to static storage, the current dynamic thermal cycling protocol introduces cyclic hydrothermal fatigue (5 °C to 55 °C) to simulate long-term intraoral thermal fluctuations. Thermal cycling was performed for 5000 (T1) and 30,000 (T2) cycles, corresponding to approximately 6 months and 3 years of clinical simulation, respectively, in accordance with the established convention of approximately 10,000 cycles per clinical year [22,23,24,25].

### 2.4. Calculation of Color and Surface Roughness Changes

Color values were measured and recorded following 5000 and 30,000 thermal cycles. The color difference between the baseline and 5000 cycles (ΔE0), and the color difference between the baseline and 30,000 cycles (ΔE1) were calculated according to the following formula:

(1) $$\Delta E^{*}\;=\;\sqrt{{\left(L2-L1\right)}^{2}+{\left(a2-a1\right)}^{2}+{\left(b2-b1\right)}^{2}}$$

The classical CIELAB formula was utilized to evaluate color alterations, adopting a clinical acceptability threshold of ΔE 3.3 to allow direct methodological comparison with previous investigations on dental PEEK and prosthetic resins [1,26].

The second surface roughness value (Ra1) was measured and recorded again at the end of 30,000 cycles. The change in surface roughness (ΔRa) was calculated and recorded as the difference between the final and initial values (Ra1–Ra0).

### 2.5. FTIR Analysis

Qualitative chemical characterization was performed via attenuated total reflectance Fourier-transform infrared (ATR-FTIR) spectroscopy on a representative specimen from each group (*n* = 1) to monitor potential functional group variations without statistical inference. FTIR spectra in the range of 4000–400 cm^−1^ were collected initially and at the end for a randomly selected specimen from each group, using a Bruker Alpha FTIR spectrometer equipped with Attenuated Total Reflectance (ATR) accessory (Bruker Optics, Ettlingen, Germany). The spectra were the results of 10 continuous scans at a resolution of 4 cm^−1^. OPUS v7.0 software was used for instrument control and spectrum manipulation [27].

### 2.6. Statistical Analysis

Statistical analyses of the study were performed using Graphpad Prism 10.6.1 (GraphPad Software Inc., Boston, MA, USA) software. The conformity of the data to normal distribution was evaluated using the Shapiro–Wilk test. Based on the results of this test, the homogeneity of variances was assessed using the Levene test. The data followed a normal distribution (*p* > 0.05). The initial roughness values (Ra0) and the roughness values after 30,000 cycles (Ra1) of the different materials were analyzed within their respective groups using Analysis of Variance (ANOVA). Additionally, one-way ANOVA was applied to analyze the ΔE0, ΔE1, and ΔRa values for each material. To determine which specific groups exhibited statistically significant differences, the Tukey HSD (Honest Significant Difference) post hoc test was utilized. The discoloration values of all groups between the T1 and T2 time intervals were evaluated using the dependent *t*-test.

## 3. Results

### 3.1. Surface Roughness

The mean and standard deviation values of the surface roughness of PEEK materials before aging and after 30,000 coffee thermal cycles are presented in Table 3. An increase in surface roughness values was observed in all groups following aging. (Ra values are presented in micrometers [µm]).

In surface roughness measurements performed after the sanding and polishing procedure applied to all specimens using the same protocol, Ra0 values exhibited statistically significant differences among materials (F(3,36) = 97.21, *p* < 0.0001). While the highest roughness values were observed in the GFP group, the TP group showed the lowest values. All groups initially exhibited Ra values below the 0.2 μm threshold (Table 3).

There was a statistically significant difference among the Ra1 values measured after 30,000 cycles (F(3,36) = 1416, *p* < 0.0001). While TP and CR exhibited roughness values below the 0.2 μm threshold, GFP and P exceeded this value (Table 3). The lowest roughness was observed in the TP group, whereas the highest roughening occurred in the GFP group.

One-way ANOVA revealed highly significant differences in surface roughness changes (ΔRa) among all groups after 30,000 cycles (F(3,36) = 10,747, *p* < 0.0001). The TP group exhibited the lowest roughness increase, whereas the GFP group demonstrated the greatest susceptibility to surface degradation, showing an approximately eightfold higher roughness change than TP (Table 3).

### 3.2. Color Stability

The mean and standard deviation values of the color change exhibited by the PEEK groups after 5000 and 30,000 cycles of coffee aging are presented in Table 4. With the increase in duration, a marked increase in color change values was observed across all groups. While the highest color change was detected in the GFP group in both periods, the highest color stability was achieved in the TP group.

Statistical analysis of ΔE0 revealed significant differences among all groups (F(3,36) = 129.9, *p* < 0.0001). The GFP exhibited the highest color change within the 5000 cycles. With the exception of this group, all other groups displayed values below the clinical threshold of 3.3 for ΔE.

Statistical analysis of ΔE1 revealed highly significant differences among all groups (F(3,36) = 703.6, *p* < 0.0001). The GFP group exhibited the highest degree of discoloration, showing a change significantly exceeding the clinical threshold. In contrast, the TP group maintained its color stability, with all specimens exhibiting ΔE1 values below the 3.3 threshold even at the end of the 30,000 cycles (Table 4).

**Figure 1:**
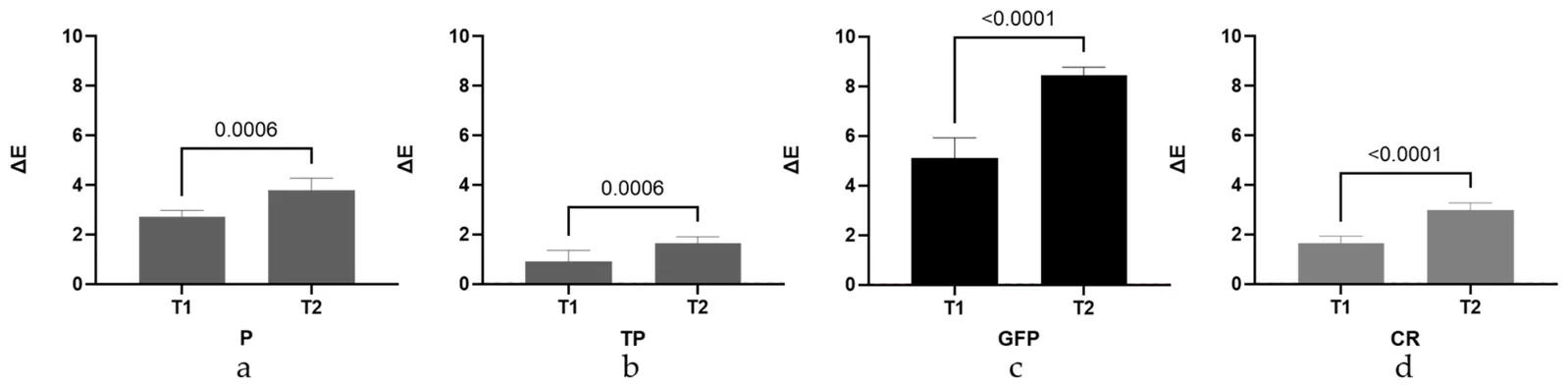
Time-dependent statistical analysis graphs. (**a**) P group; (**b**) TP group; (**c**) GFP group; and (**d**) CR group.

The time-dependent discoloration of all materials between T1 (5000 cycles) and T2 (30,000 cycles) was analyzed using paired *t*-tests (Figure 1). All groups exhibited statistically significant increases in discoloration over time (*p* < 0.0001). Specifically, paired *t*-test parameters, exact *p*-values, mean differences, and 95% confidence intervals (CI) were:

- P group: t(9) = 5.177, *p* = 0.0006, mean difference = 1.058, 95% CI [0.596, 1.520].
- TP group: t(9) = 5.196, *p* = 0.0006, mean difference = 0.739, 95% CI [0.417, 1.061].
- GFP group: t(9) = 16.48, *p* < 0.0001, mean difference = 3.349, 95% CI [2.889, 3.809].
- CR group: t(9) = 10.76, *p* < 0.0001, mean difference = 1.328, 95% CI [1.049, 1.607].

While the TP and CR groups maintained discoloration below the clinical acceptability threshold (ΔE ≤ 3.3) across both intervals, the P group exceeded this threshold at T2, and the GFP group markedly exceeded it even at T1.

### 3.3. FTIR Spectroscopy Analysis

When the FTIR spectra of all PEEK groups evaluated in the study were examined, the C=O stretching peak at 1650 cm^−1^, aromatic C=C in-ring stretching bands at 1595 cm^−1^ and 1490 cm^−1^ and C-O-C stretching bands around 1225 cm^−1^ were identified as the common and characteristic peaks of the PEEK polymer matrix.

**Figure 2:**
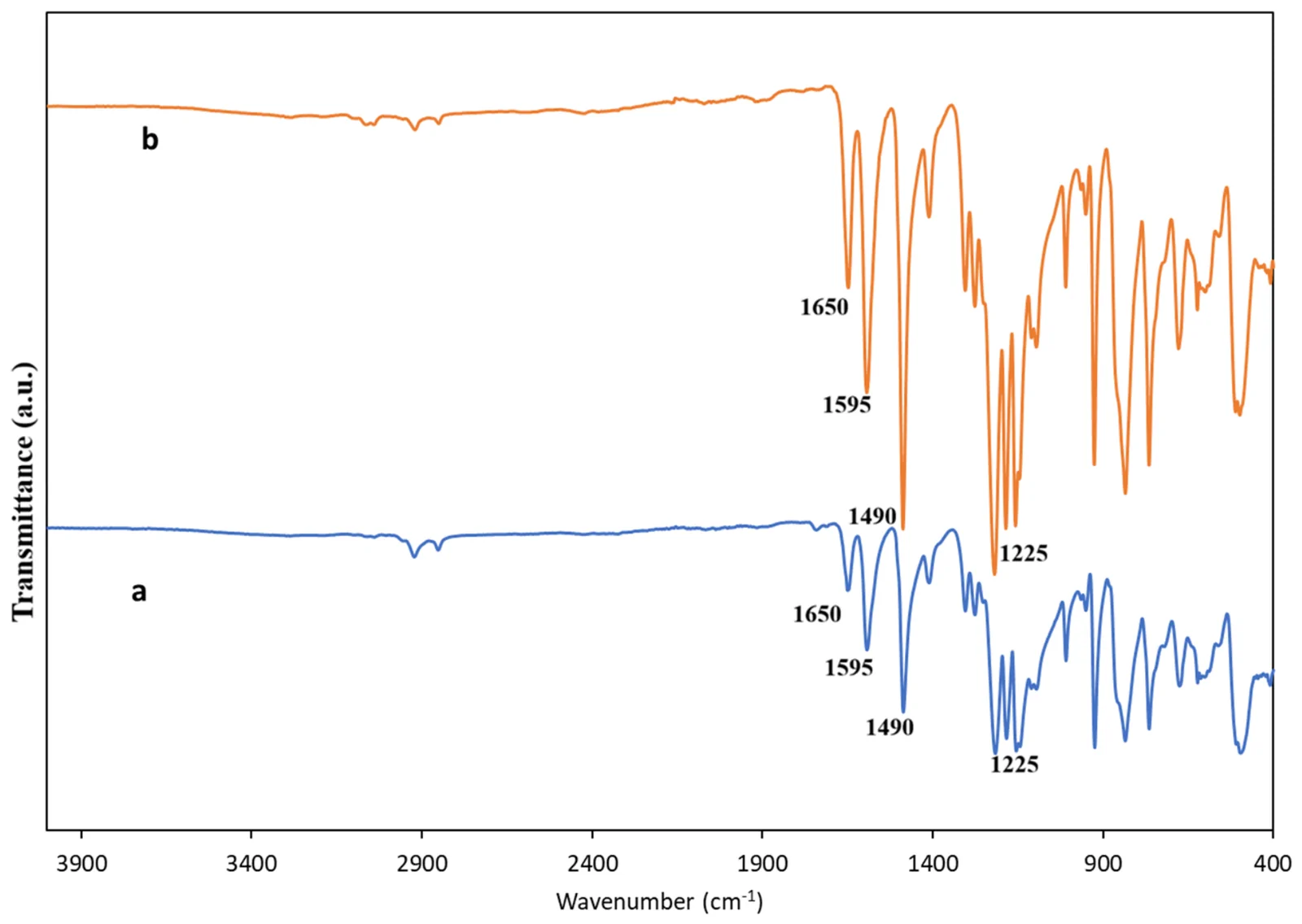
Qualitative FTIR spectra of the P material: (a) before aging; (b) after 30,000 cycles. Spectra are vertically offset along the y-axis for visual clarity; transmittance is reported in arbitrary units (a.u.). Diagnostic PEEK matrix absorption bands are labeled at 1650 cm^−^^1^ (C=O stretching), 1595 and 1490 cm^−^^1^ (aromatic in-ring C=C stretching), and 1225 cm^−^^1^ (C–O–C ether linkage).

**Figure 3:**
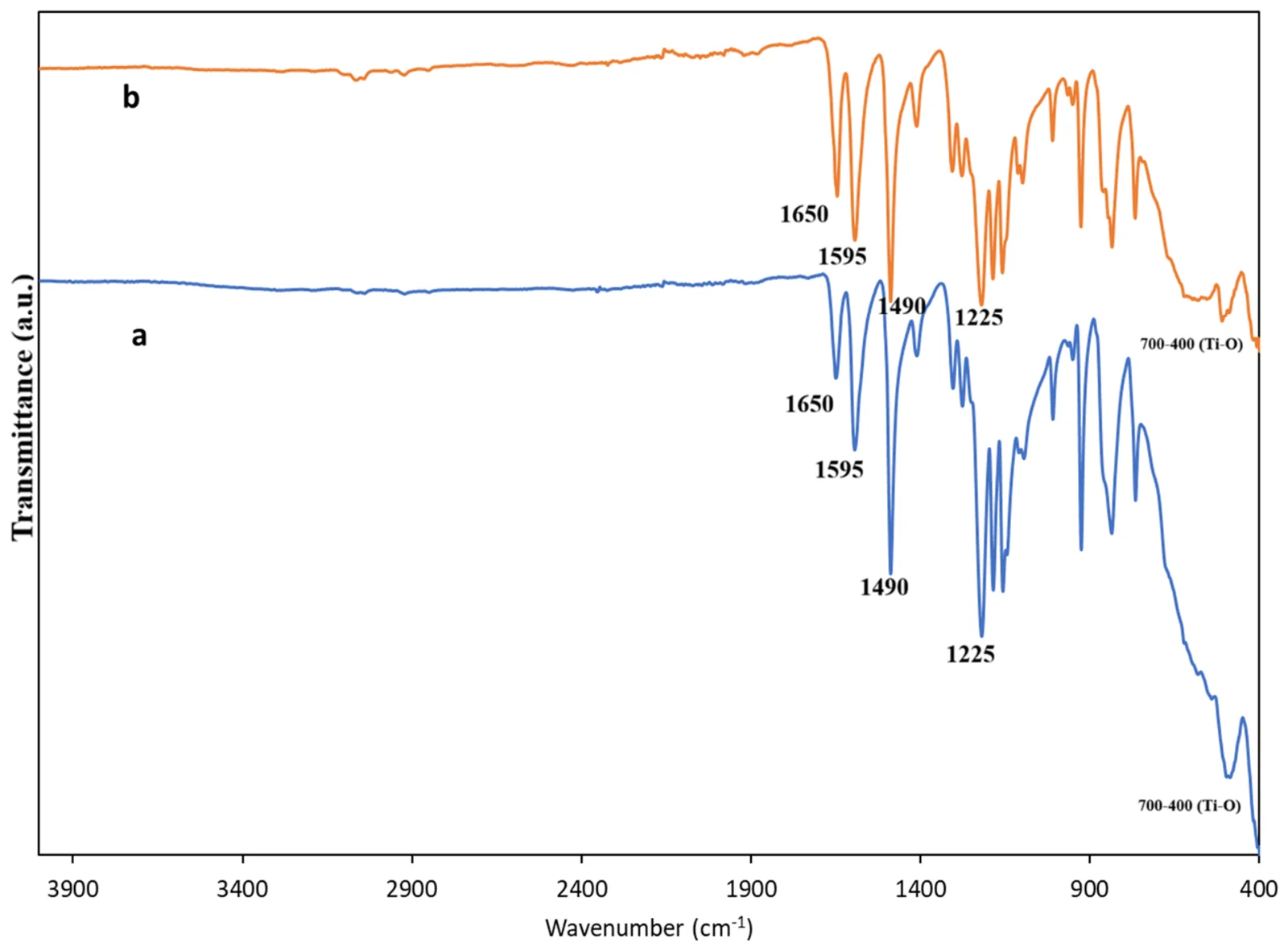
Qualitative FTIR spectra of the TP material: (a) before aging; (b) after 30,000 cycles. Spectra are vertically offset along the y-axis for visual clarity; transmittance is reported in arbitrary units (a.u.). Characteristic PEEK matrix absorption bands (1650, 1595, 1490, and 1225 cm^−1^) and the inorganic Ti–O metal-oxide lattice vibration band (700–400 cm^−1^) are labeled.

**Figure 4:**
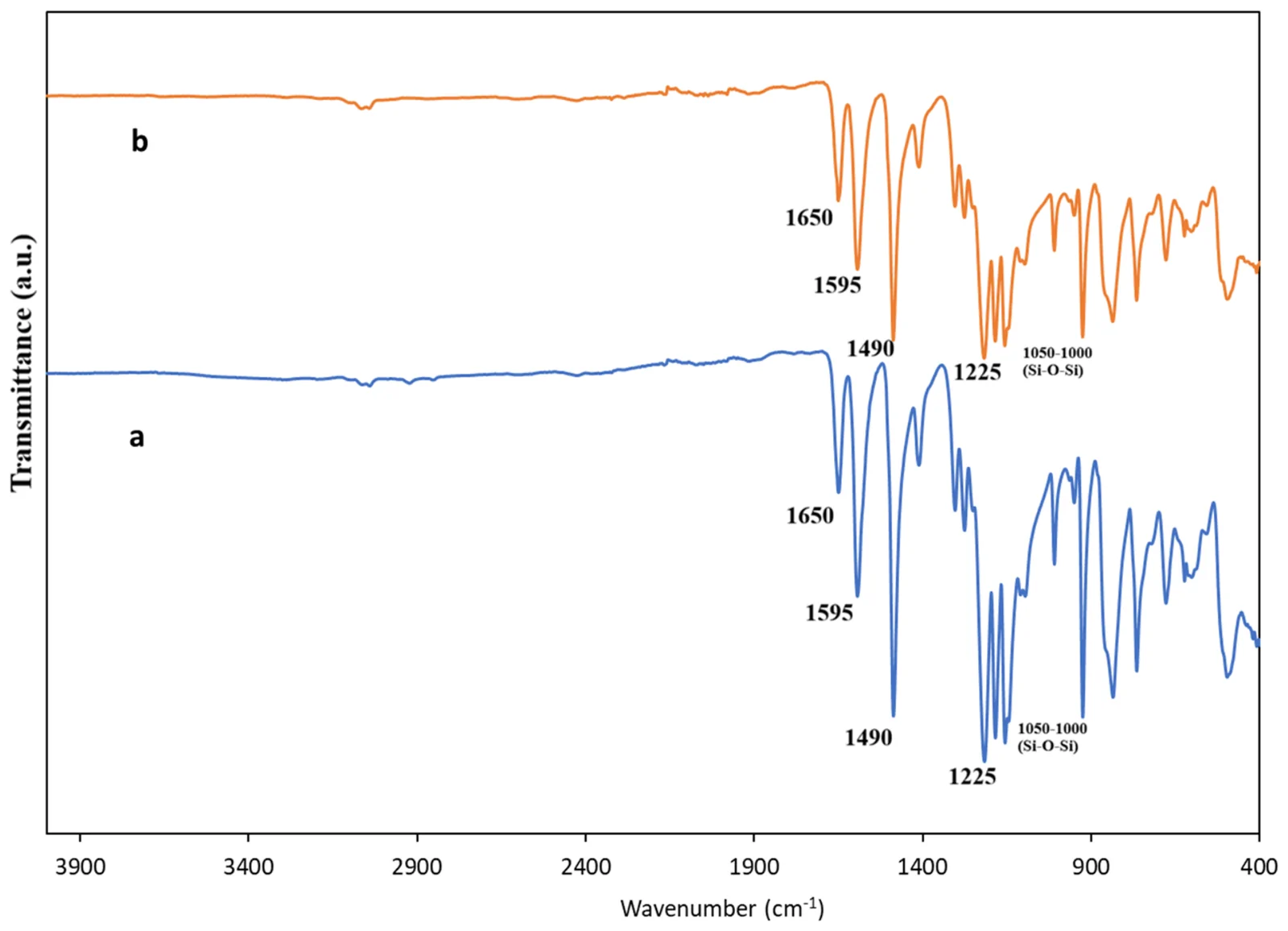
Qualitative FTIR spectra of the GFP material: (a) before aging; (b) after 30,000 cycles. Spectra are vertically offset along the y-axis for visual clarity; transmittance is reported in arbitrary units (a.u.). Characteristic PEEK matrix absorption bands (1650, 1595, 1490, and 1225 cm^−1^) and the inorganic Si–O–Si silicate stretching band (1050–1000 cm^−1^) are labeled.

**Figure 5:**
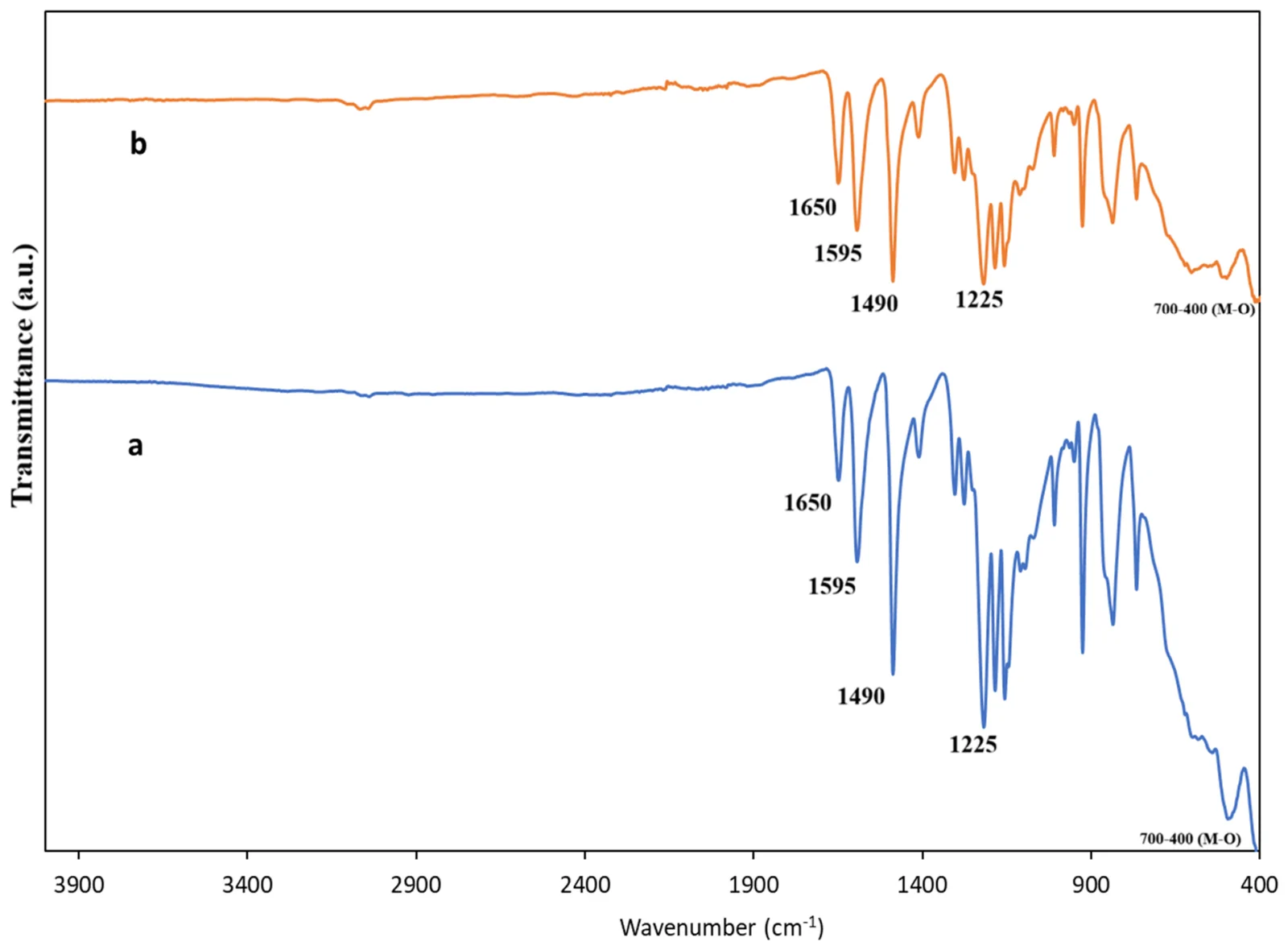
Qualitative FTIR spectra of the CR material: (a) before aging; (b) after 30,000 cycles. Spectra are vertically offset along the y-axis for visual clarity; transmittance is reported in arbitrary units (a.u.). Characteristic PEEK matrix absorption bands (1650, 1595, 1490, and 1225 cm^−1^) and inorganic ceramic metal-oxide (M–O) lattice vibration modes (700–400 cm^−1^) are labeled.

Depending on the types of fillers added to the groups, distinct structural differences were observed in the fingerprint region of 1100 cm^−1^:

- No extra band belonging to inorganic components was observed in the low wavenumber region in the P group (Figure 2).
- In the TP group, the broad absorption band observed in the 700–400 cm^−1^ region confirmed the inorganic Ti-O metal-oxide lattice vibration modes (Figure 3).
- In the GFP group, the marked broadening in the 1050–1000 cm^−1^ band confirmed the inorganic Si-O-Si stretching vibrations in the silicate structure (Figure 4).
- In the CR group, the absorption bands in the 700–400 cm^−1^ region indicated the ceramic metal-oxide (M-O) lattice vibration modes (Figure 5).

Following the 30,000 coffee thermal cycling aging protocol (Spectra b), qualitative assessment revealed no observable shifts in wavenumber (cm^−1^) positions of the main functional groups, disappearance of characteristic peaks, or new cleavage bands indicating hydrolytic degradation across the evaluated groups. Furthermore, the absence of newly formed hydroxyl (-OH) absorption bands in the 3500–3200 cm^−1^ range within ATR-FTIR sampling depth (~1–2 µm) indicates that coffee chromophores did not chemically bond to or induce hydrolytic cleavage in the polymer backbone, suggesting that discoloration was primarily mediated by superficial physical adsorption.

## 4. Discussion

Unfilled PEEK material may exhibit limitations in specific clinical applications due to its bio-inert nature and low surface energy. Ahmad et al. stated that to overcome these drawbacks, the mechanical properties and bioactivity of the material can be enhanced by incorporating titanium dioxide, ceramic particles, or carbon/glass fibers into the PEEK matrix [18]. In the literature, data regarding the surface properties and color changes in especially unfilled PEEK and reinforced PEEK materials are quite limited. The present study aims to investigate PEEK groups with different modifications to reveal the effects of these modifications on surface stability and color resistance. The findings of this in vitro study demonstrated that the CIE L∗, a∗, b∗, and Ra values of PEEK materials were significantly affected by coffee thermal cycling. Therefore, the null hypothesis that the material type and environmental conditions would not affect the color coordinates of PEEK polymers was rejected.

Thermal cycling simulates intraoral temperature changes resulting from daily diet and breathing, thereby mimicking the natural aging process of dental restorations [13]. In the study, changes occurring in the material over both short and long terms were investigated by simulating both 6-month and 3-year intraoral environments. Coffee aging and thermal cycling caused a significant increase in the surface roughness and discoloration of the materials. The clinically acceptable threshold for surface roughness in intraoral hard materials was defined by Bollen et al. as Ra 0.2 μm above which bacterial adhesion and dental plaque accumulation significantly increase [28]. Although PEEK exhibits lower surface energy and relatively hydrophobic characteristics that theoretically decrease early bacterial adherence compared to metals, recent dental literature evaluating PEEK restorative materials continues to adopt this 0.2 μm limit as the benchmark for clinical safety [29,30]. Additionally, it is noted that increased roughness levels accelerate wear in dental restorations and adversely affect long-term clinical success [31,32,33,34]. In our study, GFP and P materials exhibited surface roughness values above the threshold after 30,000 cycles. This finding suggests that further research is required regarding the long-term use of these materials.

Temperature changes occurring during thermal cycling can lead to alterations in optical properties due to internal physical and chemical transformations within the material structure. [35,36,37,38]. When individuals consume hot or cold chromogenic beverages, intraoral restorative materials undergo a dynamic thermal fluctuation process. In this regard, to investigate the changes in optical properties and surface roughness of the materials, the present study subjected them to a thermal cycling process using a coffee solution, which has a well-documented high staining potential in previous studies. [20,21,35,39]. Today, coffee is among the most consumed beverages worldwide. With average consumption rates exceeding three cups a day, coffee is considered the solution that causes the most prominent discoloration of dental materials. Some studies have scientifically shown that exposure to coffee leads to significant color degradation in restorations [40,41].

The higher increase in surface roughness observed in unfilled PEEK specimens has also been reported in previous studies. Porojan et al. stated that thermal cycling and water absorption cause degradation on the PEEK surface over time. This behavior has been attributed to the semi-crystalline structure of PEEK and its susceptibility to hydrothermal aging, which can compromise surface smoothness during long-term clinical use [42]. The observed increase in surface roughness in the unfilled PEEK group (from 0.180 to 0.234 µm) alongside unaltered ATR-FTIR spectra can be reconciled from a polymer physics perspective. In this context, ‘hydrothermal degradation’ refers to physical microstructural surface alterations rather than covalent chemical bond scission. Repetitive thermal cycling (5–55 °C) and water sorption induce superficial plasticization, localized differential thermal expansion, and micro-relief relaxation in the amorphous domains of semi-crystalline PEEK. This physical reorganization alters the polished surface topography without cleaving the chemically inert ether-ketone backbone of the polymer, consistent with our FTIR observations.

All PEEK materials used in the study initially exhibited Ra values below 0.2 μm. After 30,000 cycles, TP and CR showed roughness below the threshold value, whereas P and GFP exhibited roughness above it. Similar to our findings, the studies by Alamoush et al. and Basiony et al. observed that ceramic-reinforced PEEK was more resistant to surface modifications compared to unfilled PEEK, exhibiting lower water absorption and smaller increases in roughness [43,44]. This indicates that the ceramic filler improves the surface properties and reduces the susceptibility of the material to solutions. In studies conducted with BioHPP, it has been emphasized that it provides a bio-advantage as a denture base material and is particularly suitable for clinical use in implant-supported restorations. [45,46,47].

In our study, it is also predicted to be a material that can be used long-term in terms of color and surface roughness. In our study, the GFP group was found to be the most unsuccessful group in terms of surface and optical properties. The low resistance of the GFP group against coffee aging suggests the presence of a weak interfacial structure between the glass filler and the PEEK matrix. Tang et al. clearly state that differing polarities between the glass filler and PEEK adversely affect adhesion. Although internal porosity and water sorption were not directly measured in the present investigation, previous composite literature suggests that polarity differences and thermal expansion mismatches between inorganic glass fibers and the organic PEEK matrix can create interfacial micro-voids during thermal fluctuation [48]. These interfacial boundaries likely promoted the physical entrapment of coffee chromophores, explaining the severe discoloration and pronounced roughness increase observed in the GFP group. Fonseca et al. emphasized a strong correlation between fluid sorption and discoloration [49]. The porous structure formed in the GFP group facilitated water sorption, thereby leading to increased staining. This suggests that the intraoral use of the GFP material is limited without coating or modification. These dynamic findings correlate with and substantially expand upon our previous static immersion observations [15]. While 30 days of static immersion in coffee demonstrated noticeable surface discoloration (ΔE = 5.86 in GFP and ΔE = 3.59 in TP) without severe matrix degradation [15], the dynamic thermocycling employed in the present study generated severe hydrothermal fatigue at the filler–matrix interface. Specifically, continuous cyclic temperature fluctuations (5 °C to 55 °C) induced cyclic dimensional shifts that challenged filler–matrix cohesion, whereas static storage merely allowed passive chemical diffusion without mechanical thermal stresses. The repetitive thermal expansion mismatches between the inorganic glass fibers and the organic PEEK matrix markedly exacerbated pigment entrapment and surface roughening in the GFP group, driving discoloration up to ΔE = 8.45 and roughness change to ΔRa = 0.133 μm. Conversely, TP nanoparticles preserved both optical and topographical stability even under 30,000 dynamic cycles (ΔE = 1.66; ΔRa = 0.017 μm), demonstrating superior long-term resistance compared to static conditions.

In previous studies, titanium-reinforced PEEK material was found to be highly durable under mechanical loading; to address the gap in the literature, its surface and color changes following coffee thermal cycling were investigated [50]. Color stability is a critical aesthetic parameter, particularly for prosthetic materials used in visible areas [44]. Numerically, a ΔE ≤ 3.3 represents the clinically acceptable threshold [34,51]. In this study, all tested materials exhibited varying degrees of color degradation after coffee aging; however, titanium dioxide-reinforced PEEK demonstrated superior color stability and the lowest increase in roughness compared to other modified PEEK materials. In dental colorimetry, the classic CIELAB system and the ΔE ≤ 3.3 threshold have historically been the benchmark for clinical acceptability, representing the 50:50% visual acceptability limit where color differences become unacceptable in dental practice [1,26]. This metric was selected in the present study to maintain consistency with prior dental PEEK literature. However, modern dental research increasingly emphasizes the CIEDE2000 (ΔE00) metric due to its improved uniformity and closer alignment with human visual perception. Standardized color science thresholds established by Paravina et al. define the 50:50% perceptibility threshold (PT) and acceptability threshold (AT) as ΔE00 = 0.8 and ΔE00 = 1.8 [52]. When interpreting our findings within both frameworks, the TP group demonstrated superior color stability (ΔE = 1.66), remaining clinically acceptable under both the classical 3.3 criterion and modern rigorous thresholds. Conversely, the GFP group markedly exceeded all established clinical thresholds.

In this study, TP demonstrated superior color stability and minimal roughness increase. This finding aligns with Lu et al. [50], who evaluated commercial PEEK dental crowns with varying titanium dioxide contents and reported that titanium dioxide nanoparticles provide high chemical inertness and microstructural reinforcement. Furthermore, titanium dioxide nanoparticles enhance the opacity and brightness of the polymer, effectively masking subsurface pigment penetration and reducing the visual perception of adsorbed coffee chromophores [53]. In a similar study using different provisional materials subjected to 1000 coffee thermal cycles [54] polyethylmethacrylate, which exhibited the best color stability, showed values above the perceptibility threshold, whereas in our study, TP subjected to 30,000 cycles displayed color change below the threshold level. This serves as evidence that it can also be an alternative to other provisional crown materials. The qualitative FTIR spectroscopy findings of the present study confirmed that all PEEK variants preserved their chemical backbone integrity even after severe hydrolytic and thermal aging, such as 30,000 coffee thermal cycles. The absence of shifts in the characteristic C=O 1650 cm^−1^ and C-O-C 1225 cm^−1^ bands belonging to the PEEK matrix indicates that no chain scission occurred in the polymer chain. Furthermore, the absence of an −OH or organic component peak in the 3500–3200 cm^−1^ range proves that coffee molecules did not chemically bind to the PEEK structure and that discoloration remained purely at the superficial/physical adsorption level.

Because a CAD/CAM disc form was not available for the glass fiber–reinforced PEEK material in the study, all specimens were sectioned using a precision cutting device to ensure standardization. This factor suggests that it may have slightly increased the roughness and color change values. In the literature, it is stated that CAD/CAM milling generates fewer surface defects compared to manual methods and provides a consistent surface finish in polymers [55].

When evaluating the relationship between color stability and surface morphology, Saraswathy et al. identified a correlation between initial surface roughness and color change in PEEK material [56]. In the present study, it was observed that the increase in surface roughness occurred in parallel with the degree of discoloration. The increase in both roughness and discoloration followed a decreasing order as follows: GFP, P, CR, and TP.

This study has several limitations that should be considered when interpreting the findings. First, the applied coffee thermal cycling procedure was conducted in a closed in vitro system without continuous fluid exchange, which does not replicate dynamic intraoral variables such as salivary flow, pellicle formation, and enzymatic interactions. Second, all specimen surfaces were exposed to the staining medium, whereas intraorally only the external polished surfaces of restorations are subjected to dietary chromogens, potentially leading to higher recorded ΔE values. Third, mechanical challenges such as dynamic masticatory loading and toothbrushing abrasion—both of which directly alter surface morphology and accelerate pigment penetration—were not incorporated. Furthermore, while optical alterations were monitored longitudinally at 5000 and 30,000 cycles, surface roughness was measured only at baseline and after 30,000 cycles, precluding the assessment of intermediate degradation kinetics. Finally, chemical evaluations via ATR-FTIR were performed qualitatively on representative specimens (*n* = 1), and high-resolution microscopic techniques such as scanning electron microscopy (SEM) were not utilized to directly quantify filler distribution and interfacial wear.

To establish a more comprehensive understanding of modified PEEK biomaterials in clinical prosthodontics, future investigations should evaluate broader optical parameters, such as translucency parameter and contrast ratio, alongside varying polishing protocols. Subsequent research should also prioritize bacterial adhesion kinetics, specifically tracking *Streptococcus mutans* biofilm formation around the critical 0.2 μm Ra threshold on aged surfaces. Moreover, integrating hydrothermal aging with dynamic chewing simulators, toothbrushing wear machines, and artificial saliva storage will provide essential predictive evidence regarding the tribological, mechanical, and aesthetic longevity of these high-performance polymers under realistic oral conditions.

## 5. Conclusions

Within the limitations of this in vitro study, which evaluated the effect of coffee thermal cycling on the color stability, surface roughness, and chemical integrity of differently modified PEEK materials, the following conclusions were drawn:

1. Inorganic filler composition significantly governs the thermochemical resistance of PEEK biomaterials under dynamic aging.
2. TP exhibited superior stability, maintaining color alterations below the clinical acceptability threshold (ΔE ≤ 3.3) and surface roughness below the plaque accumulation threshold (Ra ≤ 0.2 μm) after 30,000 thermal cycles.
3. CR demonstrated acceptable surface smoothness retention (Ra < 0.2 μm) and acceptable color stability.
4. GFP was the most vulnerable material, exhibiting pronounced surface roughening (Ra > 0.2 μm) and clinically unacceptable discoloration.
5. Qualitative ATR-FTIR spectroscopy confirmed that despite physical surface topography changes, the covalent polyaryletherketone backbone remained chemically stable without hydrolytic bond cleavage across all groups.

## Figures and Tables

**Table 1:** Characteristics and manufacturers of the evaluated PEEK materials.

Materials	Content	Manufacturer
PEEK-OPTIMA™	100% PEEK (*n* = 10)	Juvora Ltd., Thornton-Cleveleys, Lancashire, UK
KERA^®^starPEEK	PEEK with 20% TiO_2_ (*n* = 10)	Main Dental GmbH, Viernheim, Germany
BioHPP	PEEK with 20% Nanoceramic (*n* = 10)	Bredent GmbH & Co.KG, Senden, Germany
TECAPEEK GF3O Naturel	PEEK with 30% Glass Fiber (*n* = 10)	Ensinger GmbH, Nufringen, Germany

**Table 2:** Study groups, material compositions, and corresponding abbreviations.

Materials	Abbreviations
100% PEEK (*n* = 10)	P
PEEK with 20% Nanoceramic (*n* = 10)	CR
PEEK with 20% TiO_2_ (*n* = 10)	TP
PEEK with 30% Glass Fiber (*n* = 10)	GFP

**Table 3:** Surface roughness values of the materials before and after aging.

Materials	*n*	Ra0 (Baseline)	Ra1 (After 30,000 Cycles)	ΔRa (Ra1–Ra0)
P	10	0.180 ± 0.006 ^b^	0.234 ± 0.007 ^b^	0.054 ± 0.003 ^b^
TP	10	0.148 ± 0.005 ^d^	0.165 ± 0.004 ^d^	0.017 ± 0.003 ^d^
GFP	10	0.190 ± 0.007 ^a^	0.323 ± 0.006 ^a^	0.133 ± 0.008 ^a^
CR	10	0.167 ± 0.005 ^c^	0.191 ± 0.006 ^c^	0.024 ± 0.003 ^c^

Different superscript lowercase letters within the same column indicate statistically significant differences among materials (*p* < 0.05, One-way ANOVA and Tukey HSD post hoc test).

**Table 4:** Color change values of PEEK groups after different aging periods.

Materials	*n*	ΔE0 (5000 Cycles)	ΔE1 (30,000 Cycles)
P	10	2.72 ± 0.25 ^b^	3.78 ± 0.49 ^b^
TP	10	0.92 ± 0.45 ^d^	1.66 ± 0.25 ^d^
GFP	10	5.10 ± 0.83 ^a^	8.45 ± 0.34 ^a^
CR	10	1.66 ± 0.27 ^c^	2.99 ± 0.28 ^c^

Different superscript lowercase letters within the same column indicate statistically significant differences among materials (*p* < 0.05, One-way ANOVA and Tukey HSD post-hoc test).

## Data Availability

The original contributions presented in this study are included in the article. Further inquiries can be directed to the corresponding author.
